# The Dimeric Peptide (KKYRYHLKPF)_2_K Shows Broad-Spectrum Antiviral Activity by Inhibiting Different Steps of Chikungunya and Zika Virus Infection

**DOI:** 10.3390/v15051168

**Published:** 2023-05-14

**Authors:** Gabriela Miranda Ayusso, Maria Letícia Duarte Lima, Paulo Ricardo da Silva Sanches, Igor Andrade Santos, Daniel Oliveira Silva Martins, Pâmela Jóyce Previdelli da Conceição, Tamara Carvalho, Vivaldo Gomes da Costa, Cíntia Bittar, Andres Merits, Norival Alves Santos-Filho, Eduardo Maffud Cilli, Ana Carolina Gomes Jardim, Marilia de Freitas Calmon, Paula Rahal

**Affiliations:** 1 Institute of Biosciences, Letters and Exact Sciences, São Paulo State University, São José do Rio Preto 15054-000, SP, Brazil; gabriela.ayusso@unesp.br (G.M.A.); maria-leticia.lima@unesp.br (M.L.D.L.); marilia.calmon@unesp.br (M.d.F.C.); 2 School of Pharmaceutical Sciences, São Paulo State University, Araraquara 14800-903, SP, Brazil; paulo.sanches@unesp.br; 3 Institute of Biomedical Sciences, Federal University of Uberlândia, Uberlândia 38408-100, MG, Brazil; 4 Laboratory of Molecular Immunology, The Rockefeller University, New York, NY 10065, USA; 5 Institute of Technology, University of Tartu, 50090 Tartu, Estonia; 6 Institute of Chemistry, São Paulo State University, Araraquara 14800-060, SP, Brazil

**Keywords:** CHIKV, ZIKV, antiviral, peptide

## Abstract

Chikungunya virus (CHIKV) and Zika virus (ZIKV) are important disease-causing agents worldwide. Currently, there are no antiviral drugs or vaccines approved to treat these viruses. However, peptides have shown great potential for new drug development. A recent study described (p-BthTX-I)_2_K [(KKYRYHLKPF)_2_K], a peptide derived from the Bothropstoxin-I toxin in the venom of the *Bothrops jararacussu* snake, showed antiviral activity against SARS-CoV-2. In this study, we assessed the activity of this peptide against CHIKV and ZIKV and its antiviral action in the different stages of the viral replication cycle in vitro. We observed that (p-BthTX-I)_2_K impaired CHIKV infection by interfering with the early steps of the viral replication cycle, reducing CHIKV entry into BHK-21 cells specifically by reducing both the attachment and internalization steps. (p-BthTX-I)_2_K also inhibited the ZIKV replicative cycle in Vero cells. The peptide protected the cells against ZIKV infection and decreased the levels of the viral RNA and the NS3 protein of this virus at viral post-entry steps. In conclusion, this study highlights the potential of the (p-BthTX-I)_2_K peptide to be a novel broad-spectrum antiviral candidate that targets different steps of the replication cycle of both CHIKV and ZIKV.

## 1. Introduction

Arboviruses constitute a group of viruses that are transmitted between vertebrate hosts via hematophagous arthropod vectors, such as ticks and mosquitoes [1]. To date, more than 500 species of arboviruses belonging to 14 different families have been reported, with more than 100 species identified as human or animal pathogens [2]. Arboviruses that cause disease in humans and animals belong to four main families: *Peribunyaviridae*, *Flaviviridae*, *Togaviridae,* and *Sedoreoviridae* [2,3]. Temperature, climate, and vegetation are ecological parameters that interfere with both vectors and host distribution [4]. Brazil is a large and tropical country with many characteristics favorable to arbovirus survival, such as climate, vegetation, and biodiversity [5]. Zika virus (ZIKV) and Chikungunya virus (CHIKV) are among the main arboviruses causing infection in Brazil [6,7].

CHIKV is an enveloped virus with a single-stranded, positive-sense RNA genome that belongs to the *Togaviridae* family and *Alphavirus* genus. This virus has a worldwide distribution and is considered a serious public health problem. According to the Pan American Health Organization (PAHO), more than 250,000 cases of CHIKV infection were reported in 12 countries and territories of the Americas in 2022. Among these countries, Brazil reported the highest incidence, with 98.8% of the cases [8]. CHIKV infection triggers symptoms in 72 to 95% of patients [9], and symptoms associated with pain and swelling, particularly in the wrists, hands, ankles, and feet, can persist for years [10]. To date, no specific antiviral therapy or licensed vaccine is available for CHIKV infection prevention or treatment, and only palliative care is recommended to alleviate the symptoms of disease [9,11].

ZIKV is an enveloped positive-sense single-stranded RNA virus. It belongs to the *Flaviviridae* family in genus *Flavivirus* [12]. ZIKV caused an epidemic in the Americas in approximately 2015, and during this period, infection in pregnant women was related to a set of neurological alterations in their fetuses named congenital Zika syndrome (CZS) [13,14,15]. ZIKV infection can also cause Guillain–Barré syndrome and other neurological complications in adults [16]. Thus, although the majority of ZIKV infection is asymptomatic, this virus is an important public health concern, especially because of CZS [15]. In 2022, more than 31,400 ZIKV infection cases were reported in the region of the Americas according to the PAHO. Most of these cases were reported in Brazil, where 92.6% of the cases were reported [8]. Similar to the case of CHIKV, there are no vaccines or antivirals approved against ZIKV [17]. Therefore, there is a significant need for treatments and vaccines that are safe and effective in reducing viral spread and limiting the disease burden caused by CHIKV and ZIKV.

In recent decades, the discovery of the therapeutic potential of several peptides with different biological targets has increased the interest of the scientific community in this class of compounds [18,19]. Peptides have cellular biocompatibility, demonstrating high affinity and specificity, which makes them potential drug candidates [18,19,20]. Antimicrobial peptides (AMPs) are derived from natural or synthetic sources. Among the synthetic peptides, there are analogs generated on the basis of natural peptide structures [18]. The peptide (p-BthTX-I)_2_ [(KKYRYHLKPFCKK)_2_], derived from the Bothropstoxin-I (BthTX-I) toxin in *Bothrops jararacussu* snake venom, has been shown to carry antibacterial activity against Gram-positive and Gram-negative bacteria [21]. Santos-Filho and coworkers demonstrated that the removal of four lysine residues located in the C-terminal region of the peptide (p-BthTX-I)_2_ [des-Lys^12^, Lys^13^-(p-BthTX-I)_2_] [(KKYRYHLKPFC)_2_] did not lead to a lack of antibacterial activity [22]. Another work reported by the same group indicated that the absence of Lys^12^ and Lys^13^, the removal of two cysteine residues, and the insertion of a lysine residue at a branch point in the C-terminal region [des-Cys^11^, Lys^12^, Lys^13^-(p-BthTX-I)_2_K–(KKYRYHLKPF)_2_K] [(p-BthTX-I)_2_K peptide) resulted in greater antibacterial activity than that of the wild-type peptide (p-BthTX-I)_2_ [23]. The synthetic peptide also showed antiviral activity against severe acute respiratory syndrome virus 2 (SARS-CoV-2), an enveloped virus that belongs to the *Coronaviridae* family [24].

This study aimed to evaluate the anti-CHIKV and anti-ZIKV activity of the (p-BthTX-I)_2_K peptide and investigate its effects on different steps of the viral infection cycle. These data constitute the first report of the antiviral activity of the (p-BthTX-I)_2_K peptide against *Alphavirus* and *Flavivirus* infections.

## 2. Materials and Methods

### 2.1. Peptide

The (p-BthTX-I)_2_K peptide [(KKYRYHLKPF)_2_K] (molecular weight = 2869.45 g/mol) was synthesized according to the method reported by Santos-Filho and coworkers [23]. In brief, the peptide was prepared on a TRIBUTE-UV automatic synthesizer (Protein Technologies, Tucson, AZ, USA) via solid phase peptide synthesis (SPPS) using the standard Fmoc protocol (9-fluorenylmethyloxycarbonyl) on a Rink-MBHA resin. To obtain the dimer, Fmoc-Lys(Fmoc)-OH was added to the C-terminus as a branch point, allowing for the growth of peptide chains from the α-amino and ε-amino groups. The peptide identity was confirmed using electrospray ionization mass spectrometry. The purity of the (p-BthTX-I)_2_K peptide was higher than 95%.

### 2.2. Cells

Baby hamster kidney fibroblast cells (BHK-21; ATCC CCL-10) and African green monkey kidney cells (Vero; ATCC CCL-81) were cultured in Dulbecco’s modified Eagle’s medium (DMEM, Cultilab, Campinas, SP, Brazil) supplemented with 10% fetal bovine serum (FBS, Gibco—Thermo Fisher Scientific, Waltham, MA, USA) and 1% penicillin (10,000 IU/mL)/streptomycin (10 mg/mL) (P/S) (Cultilab, Campinas, SP, Brazil). The cells were maintained in a humidified incubator at 37 °C in 5% CO_2_.

C6/36 cells (ATCC CRL-1660) from *Aedes albopictus* mosquitoes were cultured in Leibovitz L-15 medium (Cultilab, Campinas, SP, Brazil) supplemented with 10% FBS (Gibco—Thermo Fisher Scientific, Waltham, MA, USA) and 1% P/S (Cultilab, Campinas, SP, Brazil). These cells were maintained in an incubator at 28 °C.

### 2.3. Viruses

The recombinant CHIKV-NLuc used here was based on the CHIKV isolate LR2006OPY1 (East/Central/South African genotype). The infectious cDNA plasmid of this virus carries the human cytomegalovirus (CMV) promoter upstream of the sequence corresponding to the viral genome; the sequence of the reporter gene that encodes the NanoLuciferase (NLuc) protein was inserted into the region encoding the nsP3 protein of CHIKV [25]. ZIKV^BR^–BeH815744, isolated from a febrile patient in Paraíba state, Brazil, was used for the experiments with ZIKV [26].

### 2.4. Viral Stock Preparation

To produce infectious viral particles of CHIKV-NLuc, BHK-21 cells seeded in 24-well culture plates (TPP, Trasadingen, Switzerland) (1 × 10^5^ cells/well) were transfected with 1 µg of CMV-CHIKV-NLuc plasmid using Lipofectamine 2000 (Thermo Fisher Scientific, Waltham, MA, USA) and OPTI-MEM (reduced serum medium) (Gibco—Thermo Fisher Scientific, Waltham, MA, USA) following a previously described protocol with modifications [27]. Seventy-two hours post-transfection, the supernatant was collected and stored at −80 °C.

For preparing ZIKV stock, C6/36 cells were infected with virus and incubated for 48–96 h until a cytopathic effect was observed. The supernatant of the infected cells was collected, filtered, and stored at −80 °C.

Viral stock titers were prepared via the plaque-forming method according to the procedure used by Santos and coworkers with modifications [27]. BHK-21 cells were used for CHIKV infection, while Vero cells were used for ZIKV infection. These cells were cultured in 24-well culture plates (TPP, Trasadingen, Switzerland) (1 × 10^5^ cells/well) and infected with 10-fold serial dilutions of the respective virus for 1 h at 37 °C. Then, the viral inoculum was removed, and a semisolid medium consisting of DMEM (Cultilab, Campinas, SP, Brazil) and 2% carboxymethylcellulose (CMC) (Sigma–Aldrich, St. Louis, MO, USA) supplemented with 1% FBS (Gibco—Thermo Fisher Scientific, Waltham, MA, USA) and 1% P/S (Cultilab, Campinas, SP, Brazil) was immediately added. After incubation of the plate for 48 h (for CHIKV) and 96 h (for ZIKV), the cells were fixed with 10% formaldehyde (Merck, Darmstadt, Germany) and stained with 1% crystal violet (Merck, Darmstadt, Germany). The viral plaques were counted to determine the infectivity titer of each virus, which was expressed in plaque-forming units per milliliter (PFU/mL).

### 2.5. Cytotoxicity Analysis

The toxicity of the (p-BthTX-I)_2_K peptide in BHK-21 and Vero cells was determined using the 3-(4,5-dimethylthiazol-2-yl)-2,5-diphenyltetrazolium bromide (MTT) assay, as previously described [28] with modifications. The cells were seeded in 96-well culture plates (TPP, Trasadingen, Switzerland) (5 × 10^3^ cells/well) for 24 h. Then, the cells were incubated with the (p-BthTX-I)_2_K peptide at concentrations of 1.6, 3.1, 6.3, 12.5, 25, 50, and 100 µM. After 24 h (BHK-21) or 48 h (Vero), the medium containing the peptide was aspirated, and 100 µL of MTT (Sigma–Aldrich, St. Louis, MO, USA) diluted in DMEM (Cultilab, Campinas, SP, Brazil) (final concentration: 1 mg/mL) was added to the cells. After 30 min of incubation at 37 °C, the medium containing MTT (Sigma–Aldrich, St. Louis, MO, USA) was removed, and 100 µL of dimethylsulfoxide (DMSO) (Synth, Diadema, SP, Brazil) was added to each well of the plate, which was rotated at 200 rpm for 5 min. The absorbance was measured at a wavelength of 572 nm with a plate reader (FLUOstar Omega/BMG LABTECH, Ortenberg, Germany).

### 2.6. Analysis of the Activity of the CHIKV-NLuc-Encoded Reporter

The antiviral activity of the (p-BthTX-I)_2_K peptide against CHIKV-NLuc was determined via the measurement of the activity of the virus-encoded NLuc reporter. After incubation, the supernatant was removed, the cells were washed with PBS (phosphate-buffered saline) solution, and 30 µL of Renilla luciferase assay lysis buffer (Promega, Madison, WI, USA) was immediately added to each well. After 30 min, the plates containing the cell lysates were placed in a plate reader (FLUOstar Omega/BMG LABTECH, Ortenberg, Germany), where 50 μL of the substrate for Renilla uciferase (Renilla Luciferase assay reagent, Promega, Madison, WI, USA) was automatically injected into the wells. The light intensity was read, and the values obtained were expressed as a percentage of expression compared with that of the vehicle control (VC) (sterile water).

### 2.7. ZIKV RNA Yield Inhibition Assay Using Reverse-Transcription–Quantitative RT–PCR (qRT–PCR)

Briefly, (p-BthTX-I)_2_K antiviral activity was evaluated based on the detection of ZIKV RNA copy numbers via qRT–PCR assay. Supernatants from each well were collected at the end of every experiment, and total RNA was extracted using TRIzol reagent (Invitrogen, Waltham, MA, USA) following the manufacturer’s instructions. Viral load was then quantified using two-step qRT–PCR. The cDNA was synthesized with a high-capacity cDNA archive kit (Applied Biosystems, Waltham, MA, USA) according to the guidelines of the manufacturer. Quantification of the synthesized cDNA was then carried out using a QuantStudio™ 12 K Flex Real-Time PCR System (Applied Biosystems, Waltham, MA, USA) utilizing ZIKV 1086, ZIKV 1162c, and 1107-FAM primers [29] and TaqMan Universal PCR Master Mix, No AmpErase^®^ UNG (Thermo Fisher Scientific, Waltham, MA, USA), and 1 µL of cDNA/reaction. The thermal cycler parameters were initial denaturation at 95 °C for 10 min, followed by 40 cycles of 95 °C for 15 s and 60 °C for 1 min. The cycle threshold (C_t_) was analyzed, and the ZIKV RNA copy numbers of each sample were determined based on the standard curve and expressed on a log_10_ scale. For the post-entry assay, total RNA was isolated from infected cells. To analyze the abundance of ZIKV RNA in these samples, the amounts were normalized to the mRNA level of β-actin; the abundance of the latter was analyzed using primers (F: 5′ CAGCACAATGAAGATCAAGAT; R: 5′–CTAGAAGCATTTGCGGTGGAC, 5′–[6FAM]ACCTTCCAGCAGATGTGGATC[BHQ1]), following the ΔCt method with QuantStudio™ 12K Flex software v1.4.

### 2.8. Western Blot Analysis

To evaluate the NS3 protein of ZIKV expression in the post-entry assay, we performed a Western blot assay. The cells from the post-entry assay were collected and the cell pellet was lysed with a CelLytic M buffer (Sigma–Aldrich, St. Louis, MO, USA) containing a Protease Inhibitor Cocktail (Promega, Madison, WI, USA). The extracted proteins were quantified using the Pierce™ BCA Protein Assay Kit (Thermo Fisher Scientific, Waltham, MA, USA) to ensure equal loading in SDS-PAGE 10%. After protein denaturation, the proteins were separated in an electrophoresis system and transferred onto a polyvinylidene fluoride (PVDF) membrane (Merck, Darmstadt, Germany). After blotting, the transferred PVDF membrane was blocked for 1 h at room temperature (RT) with TBS buffer containing 5% non-fat dry milk and 0.05% Tween 20. The PVDF membrane was incubated overnight at 4 °C with rabbit polyclonal anti-ZIKV NS3 antibody (1:2000, diluted in blocking buffer), kindly provided by Dr. Andres Merit from the University of Tartu, Estonia, or rabbit anti-GAPDH monoclonal antibody (1:1000; #14C10; Cell Signaling, Danvers, MA, USA). The PVDF membrane was incubated with horseradish peroxidase (HRP)-conjugated secondary antibodies (1:5000; #31460; Thermo Scientific™, Waltham, MA, USA) for 1 h at RT. Between each reaction step, washing was performed with a TBS buffer containing 0.05% Tween 20. Finally, the target proteins were detected via ECL substrate with a ChemiDoc Imager (BioRad, Hercules, CA, USA). The band intensity of NS3 viral protein was calculated using Image Lab software (BioRad, Hercules, CA, USA) and normalized to GAPDH.

### 2.9. Primary Antiviral Analysis of (p-BthTX-I)_2_K Activity against CHIKV and ZIKV Infection

The antiviral activity of the (p-BthTX-I)_2_K peptide against CHIKV and ZIKV infection was analyzed according to a method previously described by Oliveira and coworkers [30] and Silva and collaborators [31] with modifications, respectively. For CHIKV screening, 1 × 10^4^ BHK-21 cells were seeded in the wells of 96-well white culture plates (Greiner Bio-one, Americana, SP, Brazil). For ZIKV screening, 2 × 10^4^ Vero cells were seeded in the wells of a 24-well culture plate (TPP, Trasadingen, Switzerland). Twenty-four hours later, CHIKV-NLuc or ZIKV^BR^ at a multiplicity of infection (MOI) of 0.1 and the (p-BthTX-I)_2_K peptide at the maximum nontoxic concentration (MNTC), previously established with an MTT assay, were simultaneously added to the cells. The plate was then incubated at 37 °C for 16 h (CHIKV) or 48 h (ZIKV). The inhibitory effect of (p-BthTX-I)_2_K on CHIKV was evaluated via the measurement of the NLuc activity and that of ZIKV via qRT–PCR as described above.

### 2.10. Antiviral Dose–Response Assay

The 50% effective concentration (EC_50_) for CHIKV and ZIKV inhibition was determined using the antiviral dose–response assay, as previously described [30,31] with modifications. For CHIKV, 1 × 10^4^ BHK-21 cells were seeded in the wells of 96-well white culture plates (Greiner Bio-one, Americana, SP, Brazil), and for ZIKV, 2 × 10^4^ Vero cells/well were seeded in a 24-well plate (TPP, Trasadingen, Switzerland), both 24 h before the treatment and infection. CHIKV-NLuc at an MOI of 0.1 and the (p-BthTX-I)2K peptide at a range of concentrations of 3.1, 6.3, 12.5, 25, and 50 µM were simultaneously added to the cells and incubated for 16 h at 37 °C. To ZIKV, the procedure was similar to that of CHIKV, but the concentrations were 6.3, 12.5, 25, 50, and 100 µM and the ZIKV MOI 0.1 and the peptide concentrations were incubated for 48 h. The inhibitory effect of (p-BthTX-I)2K on the CHIKV infection cycle was evaluated by measuring NLuc activity and that on ZIKV infection via qRT-PCR. The EC_50_ values were calculated using non-linear regression of the dose–response curves (Log [peptide] × response).

### 2.11. Analysis of the Protective Effect of (p-BthTX-I)_2_K against CHIKV and ZIKV Infection

The protective action of the (p-BthTX-I)_2_K peptide against CHIKV and ZIKV infection was evaluated according to the method described by Oliveira and coworkers [30] and Carneiro and collaborators [32] with modifications, respectively. The cells were seeded and incubated as described above and were then treated with (p-BthTX-I)_2_K at the MNTC. After 1 h of incubation at 37 °C, the supernatant was aspirated, the wells were washed twice with PBS, and CHIKV-NLuc or ZIKV (MOI 0.1) was added to the cells. The plate was incubated at 37 °C for 16 h (CHIKV) or 48 h (ZIKV). The inhibitory effect of (p-BthTX-I)_2_K on CHIKV infection was evaluated via the measurement of the NLuc activity and that of ZIKV via qRT–PCR as described above.

### 2.12. Investigation into the Virucidal Effect of (p-BthTX-I)_2_K

The investigation of the virucidal action of (p-BthTX-I)_2_K against CHIKV was performed following previously described methods [27,30] with modifications. For ZIKV, this assay was adapted based on a protocol described by Carneiro and collaborators [32]. For CHIKV, 1 × 10^4^ BHK-21 cells were seeded in the wells of 96-well white culture plates, and for ZIKV, 2 × 10^4^ Vero cells were seeded in the wells of 24-well culture plates (TPP, Trasadingen, Switzerland). The virions of CHIKV-NLuc at an amount to achieve infection with an MOI of 5 or those of ZIKV at an amount to achieve infection with an MOI of 0.1 were incubated with the (p-BthTX-I)_2_K peptide at the MNTC at 37 °C (CHIKV) or room temperature (ZIKV) for 1 h. Then, the viral inoculum was added to cells. After 1 h of incubation, the supernatant was removed, the cells were washed twice with PBS, and DMEM (Cultilab, Campinas, SP, BR) supplemented with 2% FBS (Gibco—Thermo Fisher Scientific, Waltham, MA, USA) was added to each well. The cells were incubated at 37 °C for the specific period previously determined for each virus. Sixteen hours post-infection (h.p.i.), CHIKV replication was quantified by measuring NLuc activity. For ZIKV, the viral RNA in the supernatant of the cells was quantified via qRT–PCR 48 h.p.i.

### 2.13. Evaluation of the Antiviral Effect of (p-BthTX-I)_2_K on CHIKV and ZIKV Entry into Cells

The antiviral activity of the (p-BthTX-I)_2_K peptide mediated by blocking viral entry into the cells was analyzed according to previously described methods [27,30,33] with modifications. BHK-21 cells (for CHIKV) and Vero cells (for ZIKV) were plated as described above. After 24 h of culture, the cells were infected with CHIKV-NLuc at an MOI of 0.1 or ZIKV^BR^ at an MOI of 0.1 in the presence of the (p-BthTX-I)_2_K peptide at the MNTC. After 1 h of incubation at 37 °C, the supernatant was aspirated, the wells were washed twice with PBS, and DMEM (Cultilab, Campinas, SP, Brazil) with 2% FBS (Gibco—Thermo Fisher Scientific, Waltham, MA, USA) was added to the cells. The plate was incubated at 37 °C for 16 h.p.i (CHIKV) or 48 h.p.i (ZIKV). (p-BthTX-I)_2_K inhibitory effects on CHIKV were evaluated via the measurement of the NLuc activity, and the effect on ZIKV was evaluated via qRT–PCR as described above.

### 2.14. Analysis of the Impact of (p-BthTX-I)_2_K on CHIKV Attachment to Cells

The antiviral effect of the (p-BthTX-I)_2_K peptide on CHIKV attachment to cell receptors was evaluated according to previously described methods [27,30,34] with modifications. In brief, 1 × 10^4^ BHK-21 cells were plated in the wells of 96-well white culture plates and cultured for 24 h. Then, the plate was incubated at 4 °C for 15 min, and CHIKV-NLuc (MOI 0.1) and the (p-BthTX-I)_2_K peptide at the MNTC were simultaneously added. The plate was again incubated at 4 °C. At this temperature, viral particles interact with cell receptors, but the virus cannot enter the cells [27]. After 1 h, the supernatant was removed, the cells were washed twice with PBS, and DMEM (Cultilab, Campinas, SP, BR) supplemented with 2% FBS (Gibco—Thermo Fisher Scientific, Waltham, MA, USA) was added. The cells were incubated at 37 °C for 16 h.p.i. The inhibitory effect of (p-BthTX-I)_2_K on CHIKV attachment to host cells was evaluated via the measurement of the NLuc activity.

### 2.15. Evaluation of the Antiviral Action of (p-BthTX-I)_2_K in CHIKV Internalization into Cells

The antiviral action of the (p-BthTX-I)_2_K peptide on CHIKV internalization into cells was analyzed according to previously described methods [34] with modifications. BHK-21 cells (1 × 10^4^ cells/well of 96-well white culture plate) were seeded 24 h before the experiment; then, the plate was incubated at 4 °C for 15 min. The cells were infected with CHIKV-NLuc (MOI 0.1). After 1 h of incubation at 4 °C, the supernatant was aspirated, and the wells were washed twice with PBS and supplemented with culture medium containing the (p-BthTX-I)_2_K peptide at the MNTC. After 1 h of incubation at 37 °C, the supernatant was removed, the wells were again washed twice with PBS, and DMEM (Cultilab, Campinas, SP, Brazil) with 2% FBS (Gibco—Thermo Fisher Scientific, Waltham, MA, USA) was added to the cells. The plate was incubated at 37 °C for 16 h h.p.i., and CHIKV inhibition was evaluated via measurement of the NLuc activity.

### 2.16. Analysis of the Antiviral Effect of (p-BthTX-I)_2_K on the Post-Entry Stages of CHIKV and ZIKV Infection

The antiviral activity of the (p-BthTX-I)_2_K peptide at the post-entry stages of viral infection was evaluated according to previously described methods [27,30] with modifications. BHK-21 and Vero cells were plated and cultured as described above and incubated with CHIKV-NLuc or ZIKV^BR^ (MOI 0.1) for 1 h at 37 °C. Then, the supernatant was aspirated, the cells were washed twice with PBS, and the (p-BthTX-I)_2_K peptide at the MNTC was added to each well. The cells were incubated at 37 °C for the period previously determined for each virus. Sixteen h.p.i. CHIKV replication was quantified via the measurement of the NLuc activity. For ZIKV^BR^, the viral RNA in the supernatant of the cells as well as intracellular viral RNA was quantified via qRT–PCR 48 h.p.i.

### 2.17. Statistical Analysis

The cytotoxicity and antiviral activity experiments were performed via three independent assays. For CHIKV, each biological replicate was performed in three (cytotoxicity) and four (antiviral activity) technical replicates; for the ZIKV assays, three (cytotoxicity) and two (antiviral activity) technical replicates were established. The statistical significance of the cytotoxic and antiviral effects of the peptide was determined using GraphPad Prism 5.0 software (GraphPad Software, San Diego, CA, USA). The statistical analyses were performed using paired Student’s t test for parametric data and Mann–Whitney test for nonparametric results. A *p* value < 0.05 was considered to be statistically significant. The data obtained were normalized to the VC and expressed as percentages.

## 3. Results

### 3.1. The (p-BthTX-I)_2_K Peptide Shows Antiviral Activity against CHIKV and ZIKV^BR^

**Figure 1:**
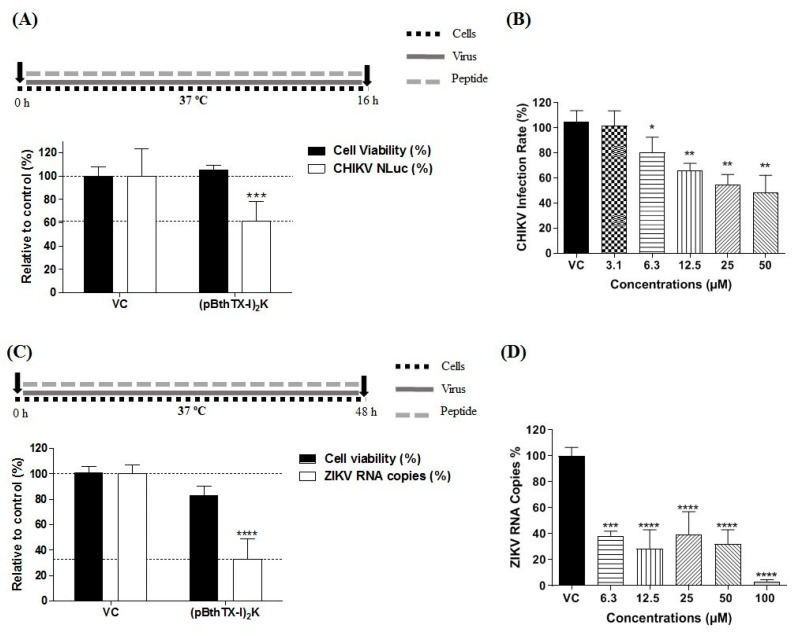
(p-BthTX-I)_2_K inhibits CHIKV and ZIKV^BR^ infection. (**A**) BHK-21 cells were incubated with (p-BthTX-I)_2_K at the MNTC (12.5 µM) or (**B**) at concentrations of 3.1, 6.3, 12.5, 25, and 50 µM, and infected with CHIKV-NLuc at an MOI of 0.1. CHIKV replication was analyzed via the measurement of the NLuc activity 16 h.p.i. Mean values  ±  standard deviations (SDs) were calculated with data obtained from a minimum of three independent experiments that were each performed in quadruplicate. (**C**) Vero cells were incubated with (p-BthTX-I)_2_K at 25 µM (the MCNT) or (**D**) at concentrations of 6.3, 12.5, 25, 50, and 100 µM and infected with ZIKV^BR^ at an MOI of 0.1. The RNA levels of ZIKV^BR^ in the cell culture supernatant 48 h.p.i. were quantified via qRT–PCR. Mean values  ± SDs represent data from three independent experiments, each of which was performed in duplicate. * *p* ≤ 0.05; ** *p* ≤ 0.01; *** *p* ≤ 0.001; **** *p* ≤ 0.0001. VC: vehicle control (sterile water). A schematic representation of the respective experiment is shown above each graph.

The MNTC of the (p-BthTX-I)_2_K peptide in BHK-21 and Vero cells was 12.5 µM and 25 µM, respectively (Figure S1A,B, Supplementary Material). When applied at the MNTC, the (p-BthTX-I)_2_K peptide reduced CHIKV replication by 38.3% (*p* ≤ 0.001) compared with the effect of the VC (Figure 1A); ZIKV^BR^ RNA levels were decreased by approximately 67.3% (*p* ≤ 0.0001) compared with those in the VC group (Figure 1C). In addition to this, the (p-BthTX-I)_2_K peptide impaired the CHIKV (Figure 1B) and ZIKV^BR^ (Figure 1D) infection in a dose-dependent manner, with EC_50_ values of 15.84 µM and 6.5 µM, respectively. Thus, the peptide significantly inhibited the infection of these two arboviruses, warranting subsequent analysis of the effects at different stages of the respective CHIKV and ZIKV infection cycle.

### 3.2. Prophylactic Effect of (p-BthTX-I)_2_K on CHIKV and ZIKV^BR^ Infection

**Figure 2:**
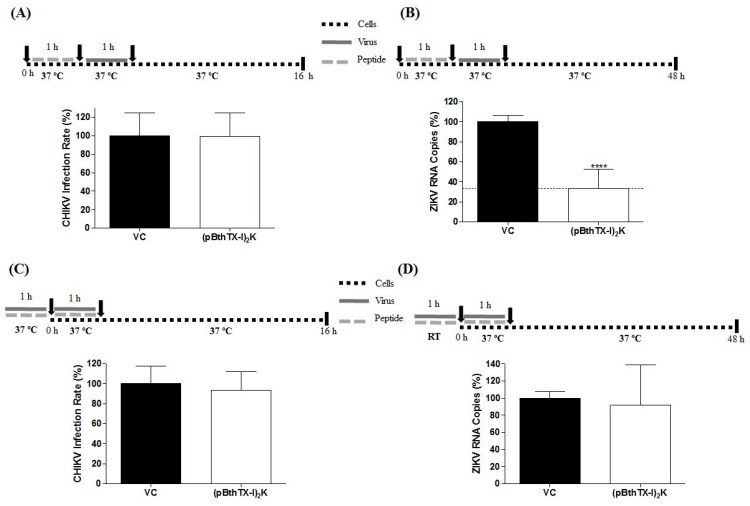
Prophylactic and virucidal activity of the (p-BthTX-I)_2_K peptide. (**A**) BHK-21 cells pretreated with the peptide at 12.5 µM showed no protection against CHIKV-NLuc infection (MOI of 0.1). (**B**) Treatment of Vero cells with peptide at 25 µM protected cells against infection with ZIKV^BR^ (MOI of 0.1). (**C**) The peptide treatment at 12.5 µM exerted no impact on CHIKV virions. (**D**) The peptide at 25 µM exerted no impact on the ZIKV^BR^ virions. CHIKV replication was analyzed via measurement of the NLuc activity 16 h.p.i. The RNA levels of ZIKV^BR^ in the cell culture supernatant were quantified via qRT–PCR 48 h.p.i. Mean values ±  SDs represent data from a minimum of three independent experiments that were each performed in quadruplicate for the CHIKV experiments and in duplicate for the ZIKV experiments. **** *p* ≤ 0.0001. VC: vehicle control (sterile water). A schematic representation of the respective experiment is shown above each graph.

To assess the protective effect of (p-BthTX-I)_2_K against CHIKV and ZIKV infection, cells were pretreated with the peptide at the MNTC. After 1 h of incubation, the cells were washed with PBS to remove the peptide and then were infected with CHIKV-NLuc or ZIKV^BR^. Pretreatment with the (p-BthTX-I)_2_K peptide did not protect BHK-21 cells against CHIKV infection (Figure 2A). However, pretreatment with this peptide protected Vero cells against ZIKV, as evidenced by the significant reduction in the ZIKV RNA levels, of 66.5% (*p* ≤ 0.0001), in treated cultures compared with VC-treated cultures (Figure 2B).

### 3.3. Virucidal Effect of the (p-BthTX-I)_2_K Peptide on CHIKV and ZIKV Extracellular Particles

To determine whether the (p-BthTX-I)_2_K peptide exerts a virucidal effect, CHIKV-NLuc and ZIKV virions were treated with the peptide at the MNTC for 1 h and then used to infect the cells. In this assay, the (p-BthTX-I)_2_K peptide did not demonstrate the ability to act on CHIKV and ZIKV virions, as indicated by its failure to reduce the NLuc levels in CHIKV-NLuc-infected cells or in ZIKV RNA levels (Figure 2C,D).

### 3.4. (p-BthTX-I)_2_K Peptide Inhibits the Early Stages of CHIKV Infection but Not the Early Stages of ZIKV Infection

**Figure 3:**
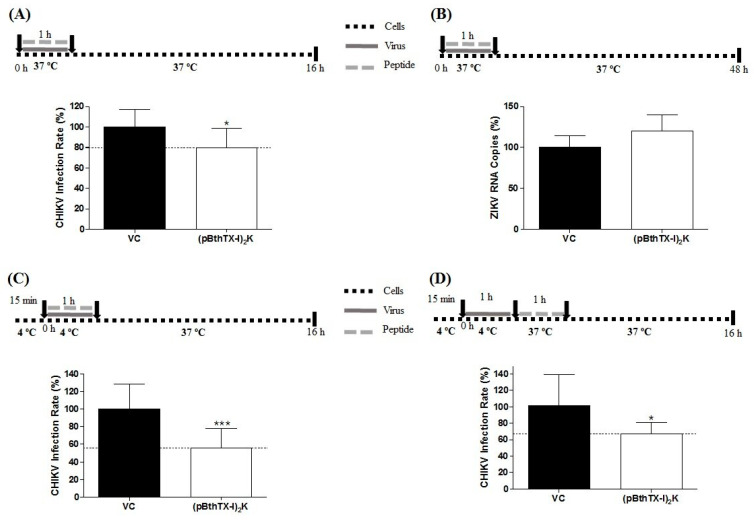
Effect of the (p-BthTX-I)_2_K peptide on the early stages of CHIKV and ZIKV^BR^ infection. (**A**) The peptide treatment at 12.5 µM significantly inhibited CHIKV-NLuc (MOI 0.1) entry into BHK-21 cells. (**B**) The peptide treatment at 25 µM did not inhibit entry of ZIKV (MOI 0.1) into Vero cells. (**C**) (p-BthTX-I)_2_K treatment at 12.5 µM exerted a significant inhibitory effect on CHIKV-NLuc attachment to BHK-21 cells. (**D**) The peptide treatment at 12.5 µM showed significant inhibitory activity on CHIKV-NLuc internalization into BHK-21 cells. CHIKV replication was analyzed via the measurement of the NLuc activity 16 h.p.i. The RNA levels of ZIKV were quantified via qRT–PCR 48 h.p.i. Error bars represent ± SD. Mean values ± SDs were calculated from the data obtained from a minimum of three independent experiments, each of which was performed in quadruplicate for the CHIKV assay and in duplicate for the ZIKV assay. * *p* ≤ 0.05; *** *p* ≤ 0.001. VC: vehicle control (sterile water). A schematic representation of the respective experiment is shown above each graph.

The action of the (p-BthTX-I)_2_K peptide on the early stages of CHIKV and ZIKV infection was evaluated. The peptide impaired CHIKV-NLuc entry into BHK-21 cells, resulting in a modest (20.3%) but significant (*p* ≤ 0.05) reduction in viral replication levels in relation to those induced in the VC group (Figure 3A). In contrast to its effect on CHIKV infection, (p-BthTX-I)_2_K did not inhibit the entry of ZIKV into cells (Figure 3B).

### 3.5. (p-BthTX-I)_2_K Peptide Affects Both the Cell Attachment and Internalization of CHIKV

The early stages of viral infection involve attachment and internalization of the virus in host cells [35]. Therefore, the antiviral effect of the (p-BthTX-I)_2_K peptide on these specific steps of CHIKV entry was investigated to determine the behavior of this peptide in the early stages of viral infection.

The (p-BthTX-I)_2_K peptide significantly inhibited CHIKV entry by reducing viral attachment to BHK-21 cells by 44% (*p* ≤ 0.001) compared with that induced by the VC (Figure 3C). Furthermore, (p-BthTX-I)_2_K significantly inhibited CHIKV internalization. At this step, the CHIKV inhibition was 34.4% (*p* ≤ 0.05) compared with that in the VC group (Figure 3D). The fact that these inhibitory effects were more prominent than those observed in the entry assay (Figure 3A) may reflect different experimental conditions (such as the use of a low-temperature step in the attachment and internalization assays).

### 3.6. (p-BthTX-I)_2_K Peptide Inhibits the Post-Entry Steps of ZIKV but Not CHIKV Infection

**Figure 4:**
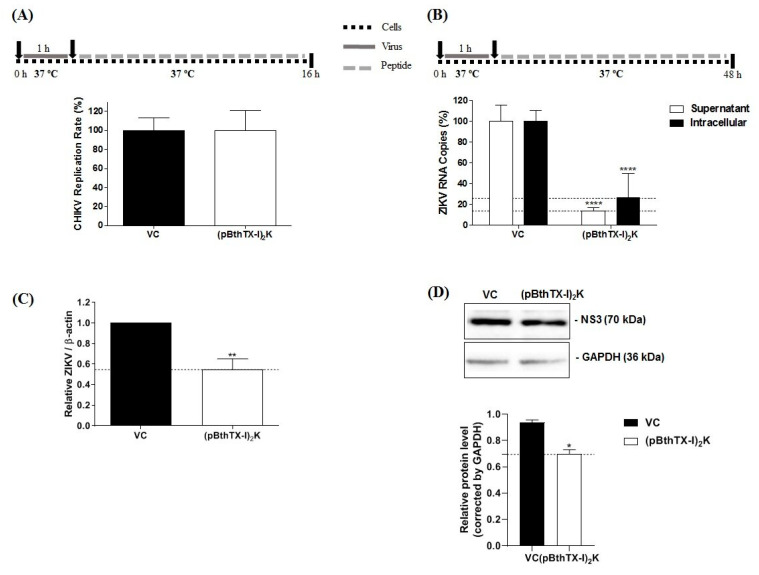
Analysis of the effect of the (p-BthTX-I)_2_K peptide on the post-entry stages of CHIKV and ZIKV infection. (**A**) Effect of peptide treatment at 12.5 µM on the post-entry steps of CHIKV-NLuc in BHK-21 cells. (**B**) Effect of the peptide treatment at 25 µM on the post-entry stages of ZIKV in Vero cells as measured by the amount of virus RNA in the supernatant and infected cells. (**C**) Normalization of ZIKV RNA levels on the basis of the ratio of the total intracellular RNA to the mRNA of β-actin. CHIKV replication was analyzed via the measurement of NLuc activity 16 h.p.i. The levels of ZIKV^BR^ RNA were quantified via qRT–PCR 48 h.p.i. (**D**) NS3 ZIKV protein expression in the post-entry assay. The level of this protein was quantified using Western blot analysis. Error bars represent ± SD. The mean values  ±  SDs represent data from a minimum of three independent experiments that were each performed in quadruplicate for the CHIKV assay and in duplicate for the ZIKV assay. * *p* ≤ 0.05; ** *p* ≤ 0.01; **** *p* ≤ 0.0001. VC: vehicle control (sterile water). A schematic representation of the respective experiment is shown above each graph.

Finally, we assessed the antiviral action of the (p-BthTX-I)_2_K peptide on the post-entry steps in the CHIKV and ZIKV infection cycles. The peptide did not exhibit an antiviral effect on the infection processes after CHIKV entry into BHK-21 cells (Figure 4A). In contrast, the peptide inhibited the post-entry steps of ZIKV infection in Vero cells: a significant difference between peptide-treated and VC-treated cells was observed. The effect was also prominent: the level of ZIKV RNA in the supernatant was reduced by 86% (*p* ≤ 0.0001), while the reduction in intracellular ZIKV RNA levels was 73.6% (*p* ≤ 0.0001) (Figure 4B). qRT–PCR analysis using β-actin mRNA as the standard confirmed that in addition to the absolute levels of intracellular ZIKV RNAs, the relative levels (normalized to the mRNA of β-actin) were significantly (*p* ≤ 0.01) reduced in the (p-BthTX-I)_2_K-treated cells (Figure 4C). Considering the study of Batista and collaborators, we sought to verify whether the effect of the peptide on ZIKV post-entry into cells may be related to the release step, and therefore, we calculated the difference in viral particles between intracellular and supernatant [28]. We observed that there was no significant reduction in viral RNA in the supernatant compared to viral RNA in the cells, indicating that (p-BthTX-I)_2_K did not act on the release step.

To better investigate the post-entry inhibition of ZIKV, we performed a Western blotting analysis to quantify the NS3 protein of this virus. The treatment of the cells after the infection by ZIKV with (p-BthTX-I)_2_K significantly reduced the level of this protein (*p* ≤ 0.05) compared with that in the cells treated with VC (Figure 4D).

## 4. Discussion

The CHIKV and ZIKV arboviruses represent an emerging health threat. Although the mortality of diseases caused by these viruses is considered to be low, the morbidity associated with CHIKV infection is high, causing social and economic impacts [36]. ZIKV infection is also a concern due to neurological alterations, such as congenital Zika syndrome and Guillain–Barré syndrome [37]. Since no antivirals or vaccines have been approved to prevent or treat these viruses, it is necessary to search for potential therapeutics [11,17].

Peptides exhibit characteristics that favor their use in therapeutic approaches, such as cellular biocompatibility and low toxicity [18,19,20]. The peptide (p-BthTX-I)_2_K is a synthetic peptide with a structure based on another peptide [(p-BthTX-I)_2_] derived from the BthTX-I toxin in snake venom, which had previously demonstrated activity against several Gram-positive and Gram-negative bacteria [23,38] and antiviral activity against SARS-CoV-2 [24], which is an enveloped positive-sense single-stranded RNA virus similar to ZIKV and CHIKV. In this study, we assessed the antiviral activity of the (p-BthTX-I)_2_K peptide against CHIKV and ZIKV infection, as well as the steps in the viral replicative cycle in which this peptide shows activity.

Our results demonstrated that this peptide reduced the infection of these viruses in a dose-dependent manner, with the antiviral effect against ZIKV being more pronounced than that against CHIKV. Notably, antiviral compounds can act at different stages of the replication cycle [39,40]. Somewhat unexpectedly, we found that the steps of the infection inhibited by the (p-BthTX-I)_2_K peptide were completely different for these viruses. The peptide inhibited CHIKV infection by interfering with the early steps of the viral replication cycle, preventing CHIKV entry into BHK-21 cells. In contrast, (p-BthTX-I)_2_K pretreatment protected cells against ZIKV, and the peptide also inhibited the post-entry steps of ZIKV infection.

The entry stage of CHIKV into host cells is divided into two steps. First, viral particles attach to the cell surface. The nonspecific interaction between cell and virus is followed by specific binding between the E2 glycoprotein present on the surface of viral particles and specific receptors on host cells, such as the cell adhesion molecule Mxra8, the main receptor of CHIKV, as well as the mucin-1 (TIM-1) protein, type 1 prohibition (PHB1) protein, and glycosaminoglycans (GAGs). This interaction triggers CHIKV internalization into cells through clathrin-mediated endocytosis. After endosome acidification, the E1 glycoprotein in the viral envelope undergoes a conformational change, resulting in the exposure of the hydrophobic fusion peptide and subsequent fusion of viral and endosomal membranes. After this process, the nucleocapsid is released into the cell cytoplasm and is disassembled, enabling the release of viral RNA [35,41]. Thus, the viral entry process involves different factors, making this step a target for several antiviral compounds [42].

We have shown that the (p-BthTX-I)_2_K peptide inhibited CHIKV entry by interfering with both attachment and internalization of the virus into BHK-21 cells. Furthermore, we observed that viral attachment was affected to a greater extent than viral internalization into these cells. We also demonstrated that the peptide did not exert an inhibitory action on CHIKV entry into BHK-21 cells by protecting the cells against viral infection or by damaging the viral particle, since this peptide did not exhibit a virucidal effect and because pretreatment of the cells provided no protection against subsequent CHIKV infection. It is not easy to explain these findings, as most compounds with an inhibitory effect on viral entry act through two main mechanisms: (1) direct action on the viral structure, inactivating virions and thus preventing their interaction with the host cell and/or (2) interference with host cell components that are crucial to viral infection [27,43].

In summary, our data showed that the (p-BthTX-I)_2_K peptide exerts its effect against CHIKV infection as a cotreatment. The inhibitory effect on viral attachment suggests that one of the antiviral modes of action for this peptide is interference of the binding of the viral glycoprotein E2 with cell receptors. One of the characteristics of peptides that favors their therapeutic use is cellular biocompatibility. Peptides are natural ligands for several cell surface receptors, demonstrating high affinity and specificity [18,19,20]. In addition, the (p-BthTX-I)_2_K peptide is a cell-penetrating peptide (CPP) [21]. Peptides in this class enter eukaryotic cells without damaging the plasma membrane. Neundorf (2019) reported that GAGs, which have been indicated to be cellular factors important for CHIKV binding, are essential for the interaction of CPPs with cells [44]. Therefore, as CPPs and CHIKV share cell receptors, competition between peptides and viruses for binding sites on the cells may explain the peptide’s antiviral effects. Most likely, the (p-BthTX-I)_2_K peptide shows greater affinity than the E2 viral glycoprotein for cell receptors, such as GAGs, reducing the number of viral particles that can enter cells.

The antiviral compounds that prevent CHIKV internalization in host cells mainly target clathrin-mediated endocytosis and endosome acidification [42]. As the main entry mechanism of CPPs into eukaryotic cells is endocytosis [44], we hypothesized that when added concomitantly to BHK-21 cells, the virus and (p-BthTX-I)_2_K peptide internalize into cells via the same endosome. Therefore, the (p-BthTX-I)_2_K peptide can exert its antiviral effects on CHIKV internalization via (1) blocking the reduction in pH in the endosomal environment, preventing the conformational change of the E1 viral glycoprotein and blocking the subsequent fusion of the viral and endosomal membranes and/or (2) interacting with the E1 viral glycoprotein, preventing the exposure of the hydrophobic fusion peptide and subsequent fusion of the viral envelope and cell membrane [39]. Finally, it is plausible that CPPs cause premature or delayed release of the virus genome into the cytoplasm, which may hamper subsequent viral infection steps.

Our work also demonstrated that the (p-BthTX-I)_2_K peptide did not cause an antiviral effect on the later stages of the CHIKV replication cycle. Therefore, this peptide did not affect the steps that follow CHIKV entry into BHK-21 cells, such as the synthesis of viral RNA, production of viral nonstructural and structural proteins, or assembly and release of viral particles [39].

Moreover, the (p-BthTX-I)_2_K peptide showed a protective effect against ZIKV infection in Vero cells but did not act on the viral entry step. Similar to CHIKV, the GAGs required for the interaction of CPPs with cells may be attachment factors for ZIKV entry into cells. However, although ZIKV and CPPs may share attachment factors [44,45], the impact of (p-BthTX-I)_2_K treatment was clearly different in CHIKV and ZIKV infection contexts. Protection against ZIKV infection required peptide pretreatment of cells; i.e., the peptide needed to be present before exposure to a virus. In the cotreatment case, the peptide was unable to inhibit viral entry, and ZIKV seemed to compete more successfully for binding cell receptors. In addition, considering that endocytosis is the main mechanism by which CPPs enter cells [44], we hypothesized that after (p-BthTX-I)_2_K pretreatment of Vero cells, the peptide was internalized via endocytosis, loaded at ZIKV post-entry sites in cells, and acted during ZIKV post-infection of cells. If this hypothesis is supported, the protective effect of pretreatment may be related to the mechanism used for the inhibition of ZIKV post-entry infection.

The post-entry stages of positive-strand RNA virus infection include genome translation, RNA replication, and virion assembly and release [46]. In summary, after the cell entry and internalization steps, ZIKV RNA is released into the cytoplasm and translated into viral proteins from the only open reading frame (ORF) in the viral RNA; the translation product is a polyprotein that is cleaved into structural and nonstructural proteins. RNA replicase is formed by nonstructural proteins associated with intracellular membranes [47,48] and binds viral RNA to initiate RNA replication. First, negative-sense RNA is synthesized, and then new positive-sense single-stranded RNAs are synthesized. Next, the new viral RNAs are packaged by structural proteins, forming immature enveloped virions budding into the endoplasmic reticulum. These virions move through the secretory pathway in the trans-Golgi network, maturing after the cleavage of the prM protein to form the M protein, and the new virions are then released from the cell via exocytosis [47,48].

A significant reduction in RNA level in the intracellular and extracellular environment of treated Vero cells may indicate that (p-BthTX-I)_2_K acts on the RNA replication steps [49]. Freire and collaborators verified that (p-BthTX-I)_2_K showed inhibitory potential against the protease PL^pro^ activity of SARS-CoV-2, and the authors suggested that peptides derived from BthTX- I, such as (p-BthTX-I)_2_K, may be useful for inhibiting the enzymatic activities of viral and cellular proteins [24]. The inhibition of these activities may be related to their effects on ZIKV post-entry steps. SARS-CoV-2 is a positive-sense single-stranded RNA which functions as mRNA that translates viral polyproteins, and viral proteases cleave these polyproteins to generate viral proteins [50]. ZIKV follows a similar process. The potential of (p-BthTX-I)_2_K to inhibit enzymatic activities, as observed with SARS-CoV-2, may be related to its effect on the post-entry steps of ZIKV infection, with the potential target being the NS2B-NS3 complex (protease) or NS5 (RNA polymerase) of ZIKV [46].

In fact, the Western blotting analysis showed a reduction in NS3 protein in treated cells, so this may be interfering with the NS2B-NS3 complex. Furthermore, the NS3 protein of ZIKV has other important rules in replication. The C-terminal domain of this protein is a helicase, which acts in the RNA synthesis unwinding the dsRNA [48,51]. This domain also has nucleoside 5′-triphosphatase, which is necessary to format the cap of the 5-terminal of the RNA [48]. The NS3 inhibition may indicate that the (p-BthTX-I)_2_K interferes with ZIKV replication.

However, the effect may also be indirect; for example, flavivirus RNA is replicated on the membranes of the endoplasmic reticulum, and (p-BthTX-I)_2_K alteration of the structure/composition/properties of this cellular compartment would exert a negative impact on ZIKV RNA replication as well. Other possible explanations include the suggestion that the peptide interferes with the viral assembly and/or maturation of virions. Our results showed that viral release did not seem to be affected by (p-BthTX-I)_2_K, as the peptide causes a similar reduction in extracellular and intracellular RNA levels. However, more studies are needed to clarify the effect of (p-BthTX-I)_2_K on the ZIKV replication cycle.

In conclusion, the data from our study showed that the (p-BthTX-I)_2_K peptide shows antiviral activity against CHIKV infection by interfering in the early stages of viral infection. The peptide inhibited viral entry into BHK-21 cells by impairing attachment and internalization into the host cell. In addition, our study demonstrated that the inhibitory action of the (p-BthTX-I)_2_K peptide on CHIKV entry was more significant on viral attachment than on internalization of the virus in BHK-21 cells. For ZIKV, this peptide caused a protective effect in Vero cells and decreased the ZIKV RNA level by inhibiting post-entry steps of the viral infection cycle. Therefore, this work suggests that the (p-BthTX-I)_2_K peptide may be a promising antiviral drug candidate against CHIKV and ZIKV, and based on its antiviral properties, suggests the use of this peptide as a template structure in the development of new broad-spectrum antiviral derivatives.

## Data Availability

Not applicable.

## Supplementary Materials

The following supporting information can be downloaded at: https://www.mdpi.com/article/10.3390/v15051168/s1. 1. Determination of the maximum non-toxic concentration (MNTC) in BHK-21 and Vero CCL-81 cells; Figure S1. Cytotoxic effect of the (pBthTX-I)_2_K peptide in (A) BHK-21 cells and (B) Vero cells. Black lines represent the arbitrary limit of 80% cell viability. VC: vehicle control (sterile water). Error bars represents ± standard deviation (SD).
