# Supplementary material for: The Dimeric Peptide (KKYRYHLKPF)2K Shows Broad-Spectrum Antiviral Activity by Inhibiting Different Steps of Chikungunya and Zika Virus Infection
Source: Viruses. 2023 May 14;15(5):1168. doi: 10.3390/v15051168 (PMC10222827; doi:10.3390/v15051168)

Supplementary Material

The Dimeric Peptide $(KKYRYHLKPF)_2K$ Shows Broad-spectrum Antiviral Activity by Inhibiting Different Steps of Chikungunya and Zika Virus Infection

Gabriela Miranda Ayusso [1†], Maria Letícia Duarte Lima [1†], Paulo Ricardo da Silva Sanches [2†], Igor Andrade Santos [3], Daniel Oliveira Silva Martins [1,3], Pâmela Jóyce Previdelli da Conceição [1], Tamara Carvalho [1], Vivaldo Gomes da Costa [1], Cíntia Bittar [1,4], Andres Merits [5], Norival Alves Santos-Filho [6], Eduardo Maffud Cilli [6], Ana Carolina Gomes Jardim [1,3], Marilia de Freitas Calmon [1], Paula Rahal [1*]

[1]Universidade Estadual Paulista (UNESP), Instituto de Biociências, Letras e Ciências Exatas, São José do Rio Preto 15054-000, SP, Brazil.

[2]Universidade Estadual Paulista (UNESP), Faculdade de Ciências Farmacêuticas, Araraquara 14800-903, SP, Brazil.

[3] Universidade Federal de Uberlândia (UFU), Uberlândia 38408-100, MG, Brazil.

[4] The Rockefeller University, New York 10065, NY, USA.

[5] Institute of Technology, University of Tartu, Nooruse 1, 50411 Tartu, Estonia.

[6] Universidade Estadual Paulista (UNESP), Instituto de Química, Araraquara 14800-060, SP, Brazil.

[†]These authors contributed equally to this work.

*** Corresponding author**

E-mail address: p.rahal@unesp.br

S1. Determination of the maximum non-toxic concentration (MNTC) in BHK-21 and Vero CCL-81 cells

The cytotoxicity of the $(pBthTX\text{-}I)_2K$ peptide was analyzed in BHK-21 cells at 24 hours or 48 hours in Vero cells by the 3-(4,5-dimethylthiazol-2-yl)-2,5-diphenyltetrazolium bromide (MTT) assay. The peptide was tested at concentrations of 1.6, 3.1, 6.3, 12.5, 25, 50 and 100 µM in BHK-21 and Vero cells. The maximum non-toxic concentration (MNTC), expressed in µM, was defined as the highest concentration of the peptide that maintained at least 80% of the cells viable within 24 hours or 48 hours. Considering the arbitrary threshold of 80% cell viability, the $(pBthTX\text{-}I)_2K$ peptide reached its MNTC in BHK-21 cells at 12.5 µM at 24 hours (Figure S1A) and 25 µM in Vero cells at 48 hours (Figure S1B).

Figure S1. Cytotoxic effect of the $(pBthTX\text{-}I)_2K$ peptide in **(A)** BHK-21 cells and **(B)** Vero cells. Black lines represent the arbitrary limit of 80% cell viability. **VC:** vehicle control (sterile water). Error bars represents ± standard deviation (SD).

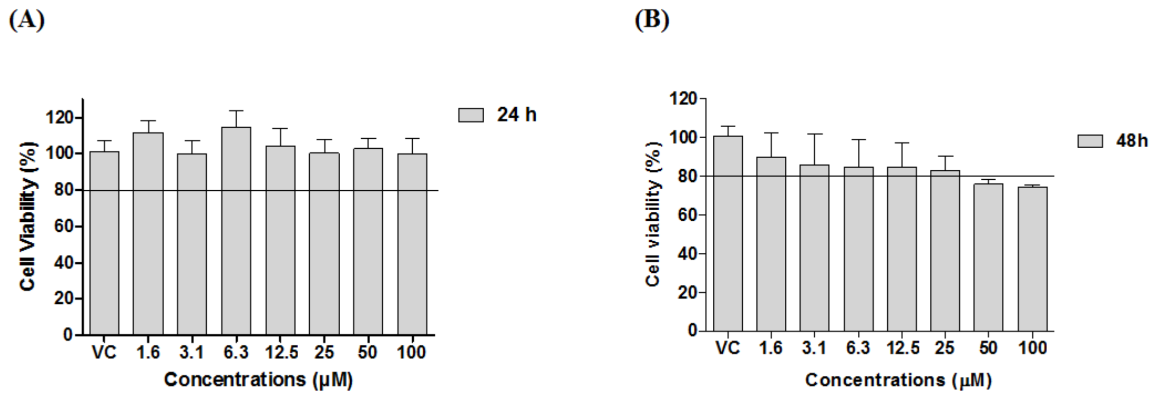

Supplement: Supplementary file 1 [file viruses-15-01168-s001.zip › viruses-2301640-supplementary.pdf]
